# Trueness of Intraoral Scanners in Implant-Supported Rehabilitations: An In Vitro Analysis on the Effect of Operators’ Experience and Implant Number

**DOI:** 10.3390/jcm10245917

**Published:** 2021-12-16

**Authors:** Paolo Pesce, Francesco Bagnasco, Nicolò Pancini, Marco Colombo, Luigi Canullo, Francesco Pera, Eriberto Bressan, Marco Annunziata, Maria Menini

**Affiliations:** 1 Department of Surgical Sciences (DISC), University of Genoa, Ospedale S. Martino, L. Rosanna Benzi 10, 16132 Genoa, Italy; 2 Department of Surgical Sciences (DISC), Division of Prosthodontics, University of Genoa, Ospedale S. Martino, L. Rosanna Benzi 10, 16132 Genoa, Italy; fcbagna5@hotmail.it (F.B.); pancini.91@gmail.com (N.P.); maria.menini@unige.it (M.M.); 3 Private Practice, 20100 Milan, Italy; m.colombo.dds@gmail.com; 4 Department of Periodontology, University of Bern, 3012 Bern, Switzerland; luigicanullo@yahoo.com; 5 CIR Dental School, Department of Surgical Sciences, University of Turin, 10126 Turin, Italy; francesco.pera@unito.it; 6 Department of Periodontology, Dental Clinic, School of Dentistry, University of Padova, 35122 Padova, Italy; eriberto.bressan@unipd.it; 7 Multidisciplinary Department of Medical-Surgical and Dental Specialties, University of Campania “Luigi Vanvitelli”, 81100 Naples, Italy; marco.annunziata@unicampania.it

**Keywords:** dental implants, digital impression, intraoral scanner

## Abstract

(1) Background: Intraoral scanners (IOS) are widely used in prosthodontics. However, a good trueness is mandatory to achieve optimal clinical results. The aim of the present in vitro study was to compare two IOS considering the operator’s experience and different implant clinical scenarios. (2) Methods: Two IOS (IT—Itero, Align Technology; and OP—Opera MC, Opera System, Monaco) were compared simulating three different clinical scenarios: single implant, two implants, and full-arch rehabilitation. Ten scans were taken for each configuration by two different operators (one expert, one inexperienced); influence of operator experience and the type of scanner used was investigated. (3) Results: Trueness of the scans differed between the experienced and non-experienced operator and this difference was statistically significant in all the three scenarios (*p* = 0.000–0.001, 0.037). A significant difference was present between the scanners (*p* = 0.000), in the two-implant and full-arch scenarios (*p* = 0.00). (4) Conclusions: Experience of the operator significantly affect trueness of IT and OP scanners. A statistically significant difference was present among IOS in the two-implant and full-arch scenarios.

## 1. Introduction

Precision and accuracy of the impression is mandatory to achieve satisfactory clinical outcomes in implant prosthodontics and different materials and techniques have been proposed to reduce possible errors during the step of data transfer to the dental laboratory [1]. In particular, over the last two decades, digital impression spread in clinical practice, in parallel with the development of computer-aided design and manufacturing (CAD/CAM), contributing to the increasingly popular digitalization of the prosthodontic workflow.

Multiple methods have been proposed to collect three-dimensional data of teeth and implants through optical cameras and laser scans [2] and numerous research have been conducted to demonstrate the reliability of these technologies.

As opposed to traditional impression, digital impressions taken with intra oral scanners (IOS) is well tolerated by the patient, since it does not require the use of conventional materials and is technically simpler for the professional [3].

Additionally, thanks to IOS the quality of the impression can be immediately verified analyzing virtual models on the computer, without producing a physical model [4]. This allows for saving time and space necessary for storing analogic models, and impressions can be sent to the dental laboratory by e-mail, eliminating shipping time and costs. Last but not least, the clinician can profit of a more effective communication with the patient together with a powerful marketing tool.

Several studies have reported high levels of accuracy and precision of IOS, both in vitro and in vivo [3,5,6,7,8,9].

Ender et al. defined the trueness as the comparison between a control STL dataset and a test STL dataset and reported that, in the case of a partial scan, the average trueness of IOS technologies is between 20 and 48 μm and the accuracy is between 4 and 16 μm compared to conventional impression, concluding that current scanners are clinically suitable for common practice [10,11,12].

On the contrary, Keul et al. compared the accuracy of five intraoral scanners to indirect digitalization using laboratory scanners and reported that direct digitalization was not superior to the indirect method [13].

A recent in vitro study analyzed the performance of two different IOS considering the operator experience. The results showed that scans of single implant rehabilitations or bridges with two pontic elements display a very high level of accuracy, in contrast with full-arch rehabilitations that presented the worst trueness [8].

The aim of the present in vitro study was to evaluate the trueness of a new recently commercialized IOS (OP—Opera MC, Opera System, Monaco) comparing it with one of the most used IOS actually available on the market (IT—Itero, Align Technology), comparing different clinical scenarios and the outcomes of operators with different clinical experience.

The null hypothesis tested was that no differences existed in trueness of intraoral scans made using different IOS, by clinicians with different learning curve and in case of different implant number.

## 2. Materials and Methods

**Figure 1:**
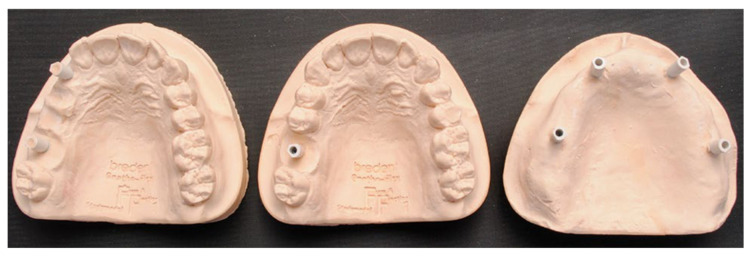
Images of the plaster models.

In the present in vitro investigation, the same methodology was applied as reported in a previously published study [8]. Three plaster master casts were made reproducing three different implant clinical situations (Figure 1):

1. Single implant in zone 16;
2. Two implants with two pontic elements (zone 13–16);
3. Full-arch rehabilitation with 4 implants (zone 13–16–23–26).

The master casts were the same used in the previously published study [8]. Scanbodies (A-INT-CAMTRA330, Sweden & Martina, Padua, Italy) were screwed on each implant analogue reproduncing a 3.30 mm Prama implant (A-ANABU-330, Sweden & Martina, Padua, Italy).

The two operators scanning the master casts were a clinician experienced with IOS (more than two years of experience with digital impression systems) and an inexperienced clinician who had never used an IOS before.

The two operators performed 10 scans for each plaster model, using the OS and the IT (total of 60 scans performed).

The “S” scan path has been applied for each scan and scanner; the tip followed the entire arch with a fluid movement starting from the last tooth of the first quadrant to the contralateral tooth while zig-zagging from vestibular to palatal and back.

To test the trueness of the scans, the three models were scanned with a reference laboratory scanner (ScanRider, V-GER) with a standard resolution of 25 to 50 μm, an average error (accuracy) of 5 to 10 μm, and a precision (standard deviation [SD]) of 15 to 30 μm. The digital impressions were then imported into a reverse engineering software (Geomagic Studio 2012, 3D Systems, Morrisville, NC, USA) and superimposed on the reference dataset. The superimposition consisted of two different procedures:

1. First, the 3-point recording function was used, where three reference points were identified on the surface of the scanbodies. This function made it possible to compare a first approximate alignment of the two models (scan deriving from the operator and reference scan) using three-dimensional (3D) surfaces.
2. The aligned models were then subjected to a cutting process with the aim of standardizing the dimensions of the different scans relative to the areas of interest around the scanbodies. A different shear pattern was used for each clinical scenario.
3. Subsequently, the best-fit algorithm was applied for the final superimposition and recording of discrepancies. With this second alignment step, after defining the reference data and parameters for registration, the polygons forming the selected models were automatically overlapped. For this final recording, a Refined Iterative Closest Point (RICP) algorithm was used, and discrepancies between the reference data and those deriving from the overlapping models were minimized using a point-to-plane method, calculating the congruence between corresponding specific structures. The same alignment parameters were used for all overlapping procedures: 100 maximum interactions and sample size set to 1000 triangles.

**Figure 2:**
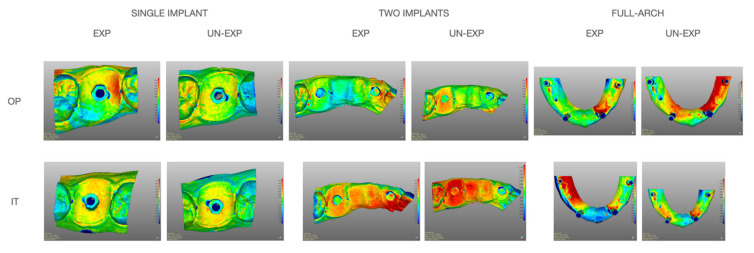
Evaluation of average deviation between RD (reference dataset) and DI (digital impression). Distances were color-coded using the 3D deviation function. IT (Itero IOS); OP (Opera system); EXP (expert operator) UN-EXP (un-experienced operator).

Distances between the corresponding reference areas and all overlapping models were color-coded using the 3D deviation function (Figure 2). Then, mean values and standard deviations were calculated.

Statistical analyses were performed with SPSS (IBM SPSS Statistics for iOS, Version 25.0. IBM Corp, Armonk, NY, USA) software.

Measurements obtained were divided into three samples based on the different clinical scenario (single implant, two implants, full-arch).

For each group, a comparison was made between the data obtained from each scanner using the non-parametric Mann–Whitney test (U-test) for two independent groups.

Subsequently, for each clinical scenario analyzed, the weight of the various independent variables analyzed (scanner, expert or un-expert operator) were analyzed with a multivariate factor analysis.

## 3. Results

Mean values of trueness comparing data deriving from the scans are reported in Table 1.

The statistical analysis revealed that the choice of the scanner was significant (*p* < 0.05) only in clinical scenarios with two implants and in full-arches (Mann–Whitney U-test).

The experience of the operator who performs the scans, on the other hand, was always significant in all the three clinical scenarios evaluated. If, on the other hand, the choice of the scanner and the experience of the operator were considered simultaneously, they were only significant in cases of full-arch.

## 4. Discussion

Currently, digital impression is widespread in implant dentistry; however, clinical outcomes might differ depending on several variables, including those considered in the present study, which are type of IOS used, operator experience and the clinical scenario. Investigations comparing such variables might be useful in order to draw specific clinical indications for different IOS systems.

The results of the present study showed that the operator experience significantly affects trueness in the three analyzed clinical conditions and the null hypothesis was, therefore, rejected. However, in the single and partial rehabilitation scenarios, the experienced clinician reported better results compared to the inexperienced clinician using both IOS, suggesting that a learning curve can improve the clinical outcomes also when applying a standardized protocol for intraoral scanning. On the contrary, in the full-arch scenario, the inexperienced operator recorded better results when using OP scanner, while trueness was greater for the experienced clinician when using IT scanner. It is also interesting to note that differences among experienced and non-expert operators increased in the full-arch scenario, that was also the configuration with the lowest levels of trueness. Such outcomes confirm that intraoral scanning might be more challenging in case of implant-supported full-arch rehabilitations compared to partial rehabilitations.

Similar results were obtained by Canullo et al. [8], where in the full-arch impression using the CS3600 (Carestream) IOS better values of trueness were obtained by the un-experienced operator.

Comparing the present outcomes with those of the previously published study, trueness mean values were better in the present study compared to the previous, except for the scans made by the inexperienced operator in the full-arch with IT. Globally, OP showed the best results. While considering the single and two-implants scenarios better results were obtained with the IT and OP scanner than with CS3660 and TRIOS3 (3shape).

Additionally, Ender et al. in 2019 [14] reported that IOS are more performing for single implants or portions of dental arch, rather than full-arch. At the same time, the results seem to suggest that some IOS perform better than others in the full-arch scenario. This is confirmed by the study by Treesh et al. of 2018 [15] reporting that in full-arch rehabilitations, the accuracy of the scans depends on the type of scanner used.

Some limits of the present research must be acknowledged. First of all, this is an in vitro study and additional difficulties that are present in the mouth were not simulated (saliva, presence of the tongue, etc.) and for this reason, the results must be taken with caution. Additionally, the use of full gypsum casts instead of using the conventional casts with pink artificial gingiva it can be a confounding factor in the algorithm capture for the intra-oral scanner. On the other hand, this in vitro protocol improves the standardization of the tests, with all the digital impression made always in the same conditions, in a repeatable and comparable way.

## 5. Conclusions

While the experience of the operator significantly affects trueness of intraoral scanners, the outcomes of the present study suggest that obtainment of optimal trueness might be more challenging in full-arch rehabilitations compared to single and partial rehabilitations. A statistically significant difference was present among IOS in the two-implant and full-arch scenarios.

## Figures and Tables

**Table 1:** Trueness (mean (SD)), in μm. Trueness decrease in the full-arch scenario (OP opera system; IT Itero IOS).

Operator	Single Implant		Two Implants		Full-Arch	
	OP	IT	OP	IT	OP	IT
Experienced	8.62 (2.13)	8.45 (1.62)	5.56 (2.29)	18.29 (3.19)	66.23 (17.53)	96.02 (12.05)
Un-experienced	10.9 (2.06)	12.59 (2.19)	7.81 (1.70)	22.01 (2.32)	39.82 (7.18)	140.84 (13.97)
*p* Mann–Whitney (scanner)	0.417		0.000		0.000	
*p* Multivariate factor analysis (operator experience)	0.000		0.001		0.037	
*p* Multivariate factor analysis (scanner)	0.269		0.000		0.000	
*p* Multivariate factor analysis (scanner and operator experience)	0.153		0.381		0.000	

## Data Availability

Data available on request.
